# Operational criteria application does not change clinicians’ opinion on the diagnosis of mental disorder: a pre- and post-intervention validity study

**DOI:** 10.3389/fpsyt.2024.1303007

**Published:** 2024-04-15

**Authors:** Helio G. Rocha Neto, José Luiz Martins Lessa, Luisa Mendez Koiller, Amanda Machado Pereira, Bianca Marques de Souza Gomes, Carlos Linhares Veloso Filho, Carlos Henrique Casado Telleria, Maria T. Cavalcanti, Diogo Telles-Correia

**Affiliations:** ^1^Programa de Pós Graduação em Psiquiatria e Saúde Mental (PROPSAM), Instituto de Psiquiatria, Universidade Federal do Rio de Janeiro (UFRJ), Rio de Janeiro, RJ, Brazil; ^2^Programa de Doutoramento do Centro Acadêmico de Medicina da Universidade de Lisboa (PhD CAML), Lisbon, Portugal; ^3^Instituto de Psiquiatria, Universidade Federal do Rio de Janeiro (UFRJ), Rio de Janeiro, RJ, Brazil; ^4^Medicine Faculty, Centro de Ciências da Saúde (CCS), Universidade Federal do Rio de Janeiro (UFRJ), Rio de Janeiro, RJ, Brazil; ^5^Clinica Universitária de Psiquiatria e Psicologia Médica, Faculdade de Medicina, Universidade de Lisboa, Lisbon, Portugal

**Keywords:** diagnosis, reliability and validity, ICD-10, mental disorders, prototype, categorical diagnosis, dimensional diagnosis, bias

## Abstract

**Objective:**

Our objective was to check if the ICD-10 operational criteria application changes non-operational, prototype-based diagnoses obtained in a real-life scenario.

**Methods:**

Psychiatry residents applied the diagnostic criteria of the ICD-10 as a “diagnostic test” to five outpatient patients they were already following who had a prototype-based diagnosis. Tests were used to ascertain whether changes in opinion were significant and if any of the diagnostic groups were more prone to change than others. The present paper is part of the study with UTN U1111-1260-1212.

**Results:**

Seventeen residents reviewed their last five case files, retrieving 85 diagnostic pairs of non-operational-based vs. operational-based diagnoses. The Stuart–Maxwell test did not indicate a significant opinion change (*χ*^2^ = 5.25, *p* = 0.39; power = 0.94) besides 30% of diagnostic changes. Despite not being statistically significant, 20.2% of all evaluations resulted in a change that would affect treatment choices. Using ICD-10 operational criteria slightly increased the number of observed diagnoses, but probably without clinical relevance. None of the non-operational diagnoses have a higher tendency to change with operational criteria application (*χ*^2^ = 11.6, *p* = 0.07). The female gender was associated with a higher diagnostic change tendency.

**Conclusion:**

Applying ICD-10 operational criteria as a diagnostic test does not induce a statistically significant diagnostic opinion change in residents and no diagnostic group seems more sensible to diagnostic change. Gender-related differences in diagnostic opinion changes might be evidence of sunk cost bias. Although not statistically significant, using operational criteria after diagnostic elaboration might help to deal with subjects without adequate treatment response.

## Introduction

### Background

Unreliability and diagnostic validity issues are a threat to both medicine and psychiatry (1–3)⁠. Clinicians’ diagnostic unreliability impairs evidence-based practice, since research participants and patients in clinical scenarios are composed of people suffering from disorders wrongly classified as the same (4)⁠. Unreliability is seen by laypeople and by scientists as a proof of low scientific evidence for clinical diagnosis, empowering political movements that see psychiatry as a way to repress society (5)⁠. It is then natural for the development of many approaches to deal with such problems (6–8)⁠.

The main approaches to reducing unreliability and improving validity in psychiatry were the development of operational-based diagnostic criteria and Standard Diagnostic Interviews (SDIs) such as “The Structured Clinical Interview for Diagnostic and Statistical Manual of Mental Disorders” (9, 10)⁠. Operational criteria are, supposedly, a pragmatic and a theoretical approach to mental disorder classification (11)⁠, similar to those used in other medical specialties such as rheumatology and cardiology (12, 13), while SDIs operate as an instrument to ensure that all psychiatric syndromes were assessed during a clinical interview. SDIs replace the individual and non-systematic observation made by clinicians through freestyle interviews, while operational criteria work as a palpable definition for mental disorders in research. The expected result of having a common measurement instrument (SDI) and a well-defined diagnostic object (operational criteria) is the improvement of both reliability and diagnostic validity.

However, SDIs are not practiced in clinical scenarios (14)⁠, and operational criteria are neither observed as part of clinical practice (3, 15, 16)⁠ nor described as how to be applied in the diagnostic process. Clinicians do not usually identify the disorder constructs described in operational criteria manuals, but rather disorder prototypes, which are nearer to clinicians’ reasoning (7, 17)⁠ and can be reasonably reliable between different clinicians (18).

The prototype diagnosis, practiced in the daily routine of clinical settings, is based on two distinct moments: the description of a disease in an “ideal kind” as defined by Weber (prototype) (19), and its identification by the clinician in the typification process (20). The prototype may contain a multitude of descriptive elements, such as signs, symptoms, measurements, and values that are ideally statistically connected, forming a comparison model for what is observed. This prototype then serves as a model for comparison by the clinician of what is observed in the patient, in a process of typification (21). After observing, collecting data, elaborating, and testing hypotheses, the clinician acquires a “model” in their mind of what the patient has, and then compares it with the different clinical prototypes they have developed throughout their training and clinical practice (22).

Clinical diagnosis was considered valid enough for clinical trials before, but it is not clear if such prototypes would fulfill operationally checked diagnosis (23). On the other hand, clinical diagnoses are sometimes considered as invalid, just because they were not obtained through an SDI, which supposedly guarantees the presence of operational criteria (23). At the same time, if clinical and research diagnoses are not equivalent, this creates a gap between research and practice, impairing evidence-based psychiatry. There are very few modern studies about diagnostic validity of non-structured, clinically based diagnosis, and the present study is an effort to understand the differences between clinical and research-based diagnosis.

Operational criteria have two effects in diagnostic validity improvement for clinical practice: First, it is a way to teach training psychiatrists how to identify a mental disorder, or at least as a scaffold for the personal prototype diagnosis development. The second role is its employment as a “diagnostic test”, to be applied after history taking and mental status examination, checking if a hypothesized diagnosis would fulfill diagnostic criteria; thus, clinically identified disorders shall be equivalent to what is practiced in research. The second option could also act as a “calibrating” step for daily practice prototypes.

### Study objectives and hypotheses

A recent brief report tested the intra-rater kappa agreement of prototype and operational-based diagnosis and has shown high reliability (16)⁠. However, that study had some limitations in checking the operational criteria power to change clinicians’ previous opinions, mainly related to the small number of clinician participants. Another limitation in the previous study concerns inherent issues in measuring kappa reliability with multiple diagnoses: mild and perhaps even moderate reliability does not rule out the possibility of significant intra-pair change differences for specific categories. Therefore, while the previous study examined diagnostic agreement before and after, this study will test if the intra-pair change is statistically significant. Obtaining consistent results using different statistical strategies and a new sample corroborates the findings obtained previously.

Our main objective in the present study is to check if clinicians identify the operational criteria in their prototype-based diagnoses, after applying operational criteria as a diagnostic test. Secondary objectives test if some prototypes are less valid, clinician bias to diagnostic change, and a “checklist effect”, where the number of observed diagnoses increases after operational criteria application (24)⁠.

Our hypothesis was that no statistically significant changes would be observed after operational criteria verification in diagnostic opinions, since clinicians would identify the criteria in their patients. As for the secondary objectives, we believed that the number of diagnostic hypotheses would not increase (checklist effect), no significant bias among clinicians would be observed, and “neurotic” [anxiety-related disorder (ARD), depressive disorder (DD), and personality disorder (PD)] prototypes would be less valid after operational criteria checking.

## Methods

### Study design

A convenience sample of patients and psychiatry trainees, in a naturalistic, real-life outpatient mental healthcare academic setting, participated in this study. IPUB is one of the leading training centers for psychiatrists in Rio de Janeiro and Brazil, with 15 psychiatrists graduating annually after 3 years of training, with a workload of 60 h per week. Residents predominantly work in outpatient clinics and inpatient management, receiving theoretical training in semiology, diagnosis, and the use of classification systems throughout the first year of training, while following up approximately 100 outpatients on monthly consultations and 30 inpatients per year. In total, residents attend to over 1,000 outpatient consultations per month and manage a 100-bed ward while under the supervision of a senior psychiatrist. By the beginning of the second year, they have completed the entire diagnostic teaching program and have been following a significant portion of their patients for over a year.

In this setting, the diagnostic practice typically follows a dynamic in which every patient, upon their first encounter with the resident assistant, undergoes a full clinical assessment comprising an unstructured history taking, psychiatric examination, and formulation of a diagnosis, even if they had already received a prior diagnosis from another attending physician. Using an SDI is not common; neither is it encouraged. The ICD-10 is the official system used in Brazil for defining and recording diagnoses in medical records, and the resident records their diagnostic hypothesis in the medical record using the respective code. However, it is not customary in daily practice to verify the presence or absence of diagnostic operational criteria, but rather to assign the code based on the identified prototype. IPUB’s outpatient functioning, Brazilian specialist training, and how prototypes are developed by residents were explained elsewhere (16, 18)⁠.

In 2022’s course, a discipline of diagnostic bias prevention was offered for the second-year residents, with an exercise of operational criteria application in previously prototype-diagnosed patients. Each participating medical resident checked their last five observed patients, and applied the ICD-10 operational criteria as a checklist, looking for differential diagnosis, following the manual inclusion and exclusion criteria, and then comparing with their previously prototypical diagnosis. These exercise results are the data used in the present study.

### Participants

Thirteen residents attending the clinical psychiatry course and four third-year residents of the research team provided their patients’ working and operationally reviewed diagnoses according to ICD-10 criteria. A hierarchical approach of the ICD-10 F chapter was applied, ensuring that only one diagnosis was considered, using the following rules: Neurodevelopmental and neurodegenerative diagnoses are persistent and affect clinical presentation for adult mental disorders (25)⁠, then subjects with a diagnosis first observed during childhood (such as autism and mental impairment) or secondary to brain damage were considered to have these diagnoses independent of further developments. A subject suffering from psychosis who not only meets the operational criteria for persistent delusional disorder, but also has operational criteria for mental impairment, would then be classified as mentally impaired. Drug use that started before other mental disorders developed was considered the main diagnosis with the other F groups. Consequently, only one diagnosis was considered before and after the ICD-10 criteria application.

### Test methods and analysis

The four-digit diagnosis was retrieved to compare the diagnoses obtained before and after the ICD-10 operational criteria application (e.g., F20.0). Those diagnoses were later converted into “main diagnosis”, considering only the three-digit category (e.g., F20), and, finally, in eight larger previously predicted groups (16)⁠: organic (diagnoses from F00 to F09), substance disorders (SD: F10–F19), schizophrenia spectrum disorders (SSD: F20–F29), bipolar affective disorder (BD: F30, F31, F34.0, and F38.1), depressive disorders (DD: F32 and F33), anxiety-related disorders (ARD: F40–F49), personality disorders (PD: F60–F69), and neurodevelopmental disorders (ND: F70–F99).

Prototype and operational-based diagnoses were paired before and after the ICD-10 operational criteria application. If a single prototype or ICD operational criteria-based diagnosis was impossible to establish, the subject was excluded from the sample. All results were pooled and then used for statistics, checking for a significant change of opinion using the Stuart–Maxwell test, a generalized version of the McNemar test.

Checking for associations of gender and prototypical diagnosis in opinion change, we created an “opinion change” measurement, where “no change” between pre- and post-operational criteria received a “0”, and a change received a “1” value. Not all diagnostic changes have the same clinical relevance (e.g., changing from a depression diagnosis to generalized anxiety disorder does not affect the prescription of antidepressant and psychotherapy, but a change from schizophrenia to post-traumatic stress disorder changes pharmacotherapy choices); thus, a “critical” measurement was created, where diagnostic changes of DD, ARD, and PD among them have attributed a value of “0”. We then used the Kruskal–Wallis test to check for a statistically significant difference for gender and prototype diagnosis in the number of diagnostic opinion changes.

Finally, to assess the presence of the “checklist effect”, we counted how many diagnostic codes the resident had used for the five patients before and after applying the operational criteria. Therefore, if, among the five observed patients, the resident made three diagnoses of severe major depression without psychotic symptoms (F32.2), one diagnosis of mania without psychotic symptoms (F30.1), and one diagnosis of mania with psychotic symptoms (F30.2), it was considered finding three diagnoses before. If this same resident, after applying the operational criteria, attributed a diagnosis of severe major depression without psychotic symptoms (F32.2), one diagnosis of mania without psychotic symptoms (F30.1), one diagnosis of paranoid schizophrenia (F20.0), one diagnosis of generalized anxiety disorder (F41.1), and another diagnosis of bipolar disorder, manic episode with psychotic symptoms (F31.1), he was considered to have found five diagnoses after applying the operational criteria. To measure if there was a statistically significant difference between the number of diagnoses before and after, indicating the presence of the checklist effect, we used the Wilcoxon test.

The Stuart–Maxwell test was run through the DescTools package of the R statistical software, and the Kruskal–Wallis and paired Wilcoxon tests were applied through R commander. As a convenience sample study, the number of prototype diagnoses in that setting was unknown, but we followed the groups previously observed in the same site (16)⁠.

IPUB’s ethics committee evaluated and approved the present study, as part of a larger diagnostic reliability study under development, registered under Certificate of Submission for Ethical Appraisal 33603220.1.0000.5263 and Universal Trial Number U1111-1260-1212, registered and approved by the Brazilian Clinical Trials Registry platform. All residents were invited to sign an informed consent, following ethical requirements for studies with human subjects. The present paper was written following STARD guidelines for diagnostic studies (26) and received no funding.

## Results

### Participants’ description

**Figure 1:**
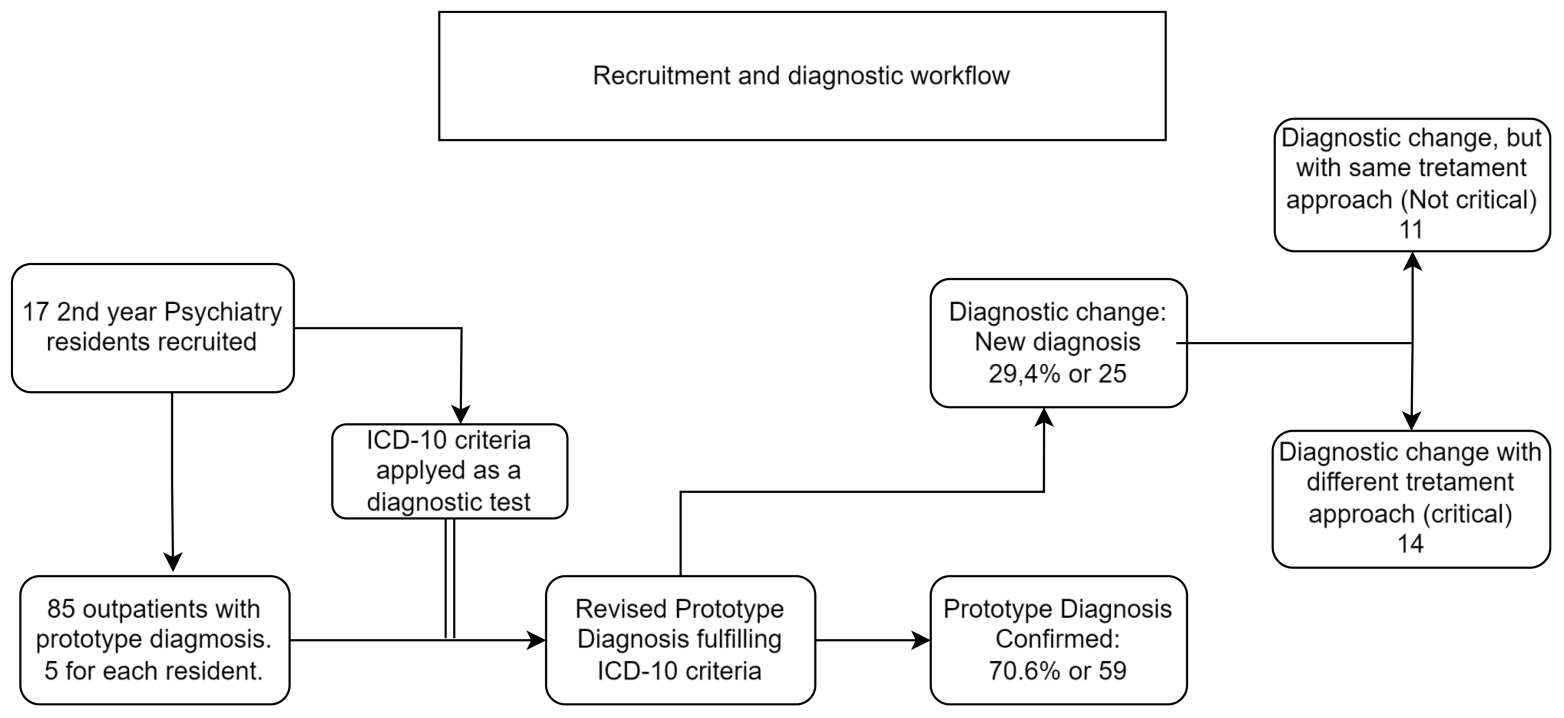
Psychiatry residents’ recruitment and diagnostic process workflow.

Seventeen residents (eight women and nine men) agreed to participate in the study, revising diagnoses from 85 patients. We did not retrieve resident demographic information other than gender, but a general picture of Brazilian residents is published elsewhere (27). A single patient that received a diagnosis of alcohol-related disorder (ICD-10 F10 category) was excluded from the Stuart–Maxwell test due to rupture of its applicability precepts, but included for the other statistics. No patient was excluded due to the impossibility of reducing diagnostic comorbidity to a single code. Patient profiles were not accessed, then their demographic information was not retrieved, but diagnostic distribution and demographic information were previously published, and the here-presented patients could be considered a small part of that sample (28). Information about the number of diagnoses identified operationally and as a prototype, with three and four digits, is described in **Table 1**. Residents considered near two differential diagnosis for each patient (mean = 1.8, SD = 0.83, range = 1–4). Retrieved diagnoses were distributed inside seven of the eight larger groups, without any representative of the “Organic” group. The diagnostic fluxogram is presented in **Figure 1**.

**Table 1:** Number of prototypes vs. ICD-10 operational-based diagnosis.

Diagnostic type		No. of diagnosis through the entire sample	Mean no. of diagnosis by resident	Wilcoxon’s *V* (no. of prototype-based vs. ICD-10-based diagnosis)	*p*	Power
All diagnoses	Prototype	38	4.58 (SD 0.51)	0	0.04*	1.00
	ICD-10	43	4.88 (SD 0.33)			
Main diagnoses	Prototype	14	4.0 (SD 0.79)	9	0.18	0.90
	ICD-10	17	4.24 (SD 0.66)			

### Test results

Residents changed their opinion in 29.4% of all cases, but the Stuart–Maxwell test did not identify statistically significant diagnostic changes after the ICD-10 criteria application (**Table 2**; *χ*^2 ^= 5.26, df = 5, *p*-value = 0.39). Starting prototype diagnosis was not associated with the number of diagnostic changes, but “female” resident gender was strongly associated with opinion changes using the Kruskal–Wallis test (**Table 3**). The use of operational criteria slightly increases the number of diagnoses considered by the residents with four-digit categories (*V* = 0, *p* = 0.04), but the difference was not significant when categories collapsed to three digits (*V* = 9, *p* = 0.18).

**Table 2:** Prototype of large groups vs. ICD-10 criteria-checked large group diagnoses.

Prototype-based diagnoses	ICD-10 criteria-checked diagnoses							Frequency of diagnostic opinion change
	ARD	BD	DD	ND	PD	SSD	Total	
ARD	**10**	0	1	2	1	0	14	29%
BD	1	**9**	1	0	1	1	13	31%
DD	2	3	**5**	0	2	0	12	58%
ND	1	0	0	**1**	0	0	2	50%
PD	3	2	2	0	**15**	1	23	35%
SSD	0	0	0	1	0	**19**	20	5%
Total	17	14	9	4	19	21	84	29.4%
Stuart–Maxwell test: χ^2^ = 5.25, p = 0.39; power = 0.94. ARD, anxiety-related disorders; BD, bipolar affect disorder; DD, depressive disorder; SSD, schizophrenia spectrum disorder; ND, neurodevelopmental disorder; PD, personality disorder. Numbers in bold represents diagnostic agreement of before and after ICD-10´s operational criteria checked diagnosis.								Critical changes: 20.2% Critical/general changes ratio: 56% Don’t change/change odds: 2.36

**Table 3:** Diagnostic opinion change associations.

	Kruskal–Wallis *χ*^2^	Df	*p*-value	eta²
Prototype				
ICD-10 4dig Diagnoses	40.46	38	0.36	–
ICD-10 3dig Diagnoses	17.28	14	0.24	–
ICD-10 Group Diagnoses	11.6	6	0.07	–
ICD-10 Group Diagnoses Critical change	3.48	6	0.75	–
Gender				
ICD-10 4dig Diagnoses	7.15	1	0.01*	0.07
ICD-10 3dig Diagnoses	7,16	1	0.007**	0.07
ICD-10 Group Diagnoses	6,16	1	0.01*	0.06
ICD-10 Critical change	10.5	1	0.001**	0.11

* p<0.05, **p<0.01.

## Discussion

Using the ICD-10 operational criteria for diagnostic validation did not statistically change clinician opinion, corroborating with our initial hypothesis and a previous study with the same objective (16). However, these findings do not mean that operational criteria application is useless for diagnostic validation, nor that these changes are irrelevant, since one in each of the five checked diagnoses changed between critical groups, which might cause clinical treatment revision (**Table 2**). In addition, our findings suggest that operational criteria use may be particularly relevant for differential diagnosis in subjects with initial prototype hypothesis of DD, being relevant for both daily clinical training and treatment revision for patients who do not have shown clinical improvement.

One possible explanation for the lack of detection of differences by the Stewart–Maxwell test could be a failure to obtain a sample of adequate size. Although the post-hoc test suggests that the sample used has sufficient power to detect statistical differences for moderate size effect in a comparison with six variables, two issues need to be considered: different validities between groups and problems with post-hoc tests. In the first case, **Table 3** shows that the Kruskal–Wallis test does not identify a significant difference among the seven groups, but with a *p*-value (0.07) indicating that a larger sample could reach the critical value. When diagnoses are grouped into six “critical” ones, the *p*-value changes dramatically (0.75). The significant difference here is that, in the second test, changes between ARD, PD, and DD are treated as absence of change, which possibly indicates that these groups change significantly among themselves but minimally for other diagnoses. Additionally, the calculation of post-hoc values is highly dependent on the *p*-value, which can inflate the power calculation in a small sample with non-significant results.

Against previous findings (16)⁠, a “checklist effect” was detected, with an increase in diagnostic variety after operational criteria application. Despite that, this effect was not statistically relevant with the fourth ICD-10 digit suppression, decreasing diagnostic options to the larger diagnostic group that usually guides treatment decisions (e.g., F32.2 to F32) (**Table 1**). Moreover, the number of ICD-10, operational-based total used diagnoses dropped from 43 to 17 after fourth-digit suppression, and the remaining 17 could be grouped into eight large groups of diagnosis prototypes, suggesting that the number of relevant diagnoses for clinical practice might be lower than presented in diagnostic manuals (7).

Except for the previously discussed possibility that ARD, DD, and PD may exhibit validity issues among themselves, we did not find evidence of a prototype diagnostic group more prone to change (less valid) after operational criteria application, independently of the number of possible diagnoses considered (**Table 3**). In our opinion, this is evidence of the prototype diagnosis validity, at least in intra-rater evaluations. However, clinician gender affects the probability of diagnostic opinion change after operational criteria application, raising an alert for possible bias in those observations.

There is no reason to consider that female residents would formulate less valid prototypical diagnoses when compared with men. However, women have a higher tendency to follow and apply guidelines in medicine (29, 30)⁠, and men are overconfident in general, which might hinder their ability to change (31, 32)⁠. These characteristics may reflect a higher predisposition for women to consider new information and change their opinion when presented with more evidence, while men might resist changing their diagnostic opinion. If this is true, gender influence in diagnostic changes might reflect a “sunk cost bias”, with unknown consequences for reliability and validity studies.

Sunk cost bias is the name of a natural difficulty to change previous opinion/diagnosis, especially due to the need to accept a previously wrong or badly made decision (33). Despite female patients’ tendency to change, we could not say that they are immune to such bias, so the impact of sunk cost is probably greater than presented here. If the clinical diagnostic opinion is seldom prone to change after conclusion, improving the ability to identify the right prototypes must go beyond an operational criteria checklist applied after evaluation. An interesting approach shall be applying a standard approach to history taking and mental status examination, although they are unavailable nowadays (34, 35)⁠.

### Study limitations and future research

The present study has the following limitations: First of all, its design aimed to verify diagnostic opinion change before and after operational criteria application; thus, secondary findings shall be observed cautiously. In the same way, clinicians were asked to review five patients’ diagnoses, creating a ceiling effect for the number of possible differently observed and identified diagnoses by each subject and restraining the observable diagnostic variability. It is possible that if each clinician brought 10 reviewed diagnoses, the number of four- and three-digit diagnoses would have been greater, as it was in other studies (16)⁠, improving the ability to verify the checklist effect, although it is unlikely that the final list of large diagnostic groups would be different. The present paper also uses data generated during a psychiatry clinical course, implying some clinical inexperience from clinicians, and an unknown adherence to operational criteria application as requested. Another limitation lies in the caution with which our data should be observed: similar to the previous study, the psychiatry residents comprising this sample are all students from the same teaching and assistance service. IPUB is considered a reference institution in the training of psychiatrists in Brazil and follows the rules for specialist training that should be replicated throughout the country. However, it is possible that training in other locations, especially abroad, may lead to different formats for conducting interviews and reaching diagnostic conclusions, for example, using a structured tool or requiring operational criteria to be checked off in a checklist during the initial interview. Although it is not clear in the literature how operational criteria should be used, their irregular use may be a local (Brazilian) phenomenon or one found in some, but not all, other countries.

Our results are a seed for future larger and better-funded studies, which could recruit more experienced clinicians in real-life scenarios and use our findings for sample size calculation to test the validity of prototype diagnosis. Furthermore, we did not address information, anchoring, and other data-collection-related biases that could have changed diagnostic opinion after providing more clinical information to doctors. A study comparing SDI and clinically based diagnosis might help understand the size of these biases in diagnostic validity.

## Conclusion

In the present scenario and extracted data, operational criteria application had no statistically relevant influence on diagnostic opinion change, confirming our initial hypothesis of prototype diagnosis validity, but might be a key instrument to improve diagnostic evaluation in patients that do not respond to treatment. The checklist effect might exist in a larger sample, which was not our initial hypothesis; however, its size effect is probably small, and it may not be relevant for clinical practice. Against our hypothesis, no prototypical diagnoses were statistically more vulnerable to change after operational criteria checking, even considering that most initial diagnoses of depression were changed. Gender association to diagnostic opinion change was not expected, and we hypothesize that it is an indication of sunk cost bias after setting a diagnostic opinion, a problem yet to be better understood in reliability studies.

## Data availability statement

The original contributions presented in the study are included in the article/supplementary material. Further inquiries can be directed to the corresponding author.

## Ethics statement

The studies involving humans were approved by Comite de Ética em Pesquisa do Instituto de Psiquiatria da UFRJ. The studies were conducted in accordance with the local legislation and institutional requirements. The participants provided their written informed consent to participate in this study.

## Author contributions

HGRN: Conceptualization, Data curation, Formal analysis, Investigation, Methodology, Project administration, Writing – original draft, Writing – review & editing. JL: Investigation, Project administration, Supervision, Validation, Visualization, Writing – review & editing. LK: Data curation, Investigation, Validation, Visualization, Writing – review & editing. AP: Data curation, Investigation, Visualization, Writing – review & editing. BM: Data curation, Investigation, Validation, Visualization, Writing – review & editing. CV: Data curation, Investigation, Validation, Visualization, Writing – review & editing. CHCT: Investigation, Validation, Visualization, Writing – review & editing. MC: Investigation, Project administration, Supervision, Validation, Visualization, Writing – review & editing. DT-C: Supervision, Validation, Visualization, Writing – review & editing.
